# Supplementary material for: A Comprehensive Assessment of Ultraviolet-Radiation-Induced Mutations in Flammulina filiformis Using Whole-Genome Resequencing
Source: J Fungi (Basel). 2024 Mar 20;10(3):228. doi: 10.3390/jof10030228 (PMC10971301; doi:10.3390/jof10030228)
Supplement: Supplementary file 1 [file jof-10-00228-s001.zip › Supplementary Material S8/KEGG annotation/out/64381550635650.os/KO/out_map/map04728.html]

KEGG PATHWAY: Dopaminergic synapse - Reference pathway


|  |  |
| --- | --- |
| **Dopaminergic synapse - Reference pathway** |  |

[
Pathway menu
| Organism menu
| Pathway entry
| Show description
| User data mapping
]

Dopamine (DA) is an important and prototypical slow neurotransmitter in the mammalian brain, where it controls a variety of functions including locomotor activity, motivation and reward, learning and memory, and endocrine regulation. Once released from presynaptic axonal terminals, DA interacts with at least five receptor subtypes in the central nervous system (CNS), which have been divided into two groups: the D1-like receptors (D1Rs), comprising D1 and D5 receptors, both positively coupled to adenylyl cyclase and cAMP production, and the D2-like receptors (D2Rs), comprising D2, D3, and D4 receptors, whose activation results in inhibition of adenylyl cyclase and suppression of cAMP production. In addition, D1Rs and D2Rs modulate intracellular Ca2+ levels and a number of Ca2+ -dependent intracellular signaling processes. Through diverse cAMP- and Ca2+-dependent and - independent mechanisms, DA influences neuronal activity, synaptic plasticity, and behavior. Presynaptically localized D2Rs regulate synthesis and release of DA as the main autoreceptor of the dopaminergic system.

|  |  |
| --- | --- |
| Reference pathway | 100% |
